# Effects of Leader Conscientiousness and Ethical Leadership on Employee Turnover Intention: The Mediating Role of Individual Ethical Climate and Emotional Exhaustion

**DOI:** 10.3390/ijerph19158959

**Published:** 2022-07-23

**Authors:** Tajneen Affnaan Saleh, Abdullah Sarwar, Md. Amirul Islam, Muhammad Mohiuddin, Zhan Su

**Affiliations:** 1 Faculty of Management, Malaysia Multimedia University, Cyberjaya 63100, Malaysia; tazneenafn@gmail.com (T.A.S.); abdullah.sarwar@mmu.edu.my (A.S.); amirulmiu@gmail.com (M.A.I.); 2 Faculty of Business Administration, Lval University, Quebec, QC G1V 0A6, Canada; zhan.su@fsa.ulaval.ca

**Keywords:** leader conscientiousness, ethical leadership, emotional exhaustion, individual-type ethical climate, turnover intention

## Abstract

Employees working under conscientious leadership perceive their leaders as ethical leaders. This study investigates the conscientiousness of leaders as an essential trait of ethical leadership and the relationship between ethical leadership and employee-turnover intention. Additionally, we study the potential mediating roles of the individual-level ethical climate (self-interest, friendship, and personal morality) as well as the level of employees’ emotional exhaustion that contribute to the decision-making process of turnover intention. Building on social learning and social exchange theories, outcomes from nine industrial manufacturing organizations comprising 260 subordinates’ responses show that leaders’ conscientiousness is positively related to ethical leadership and negatively associated with employees’ turnover intention. Consistent with this hypotheses, results found that, in an individual-level ethical climate, employees experience diminished emotional exhaustion. The relationships are found to mediate between ethical leadership and turnover intention in manufacturing organizations. Additionally, it was also found that individual-level ethical climates cause a relatively positive impact on employees’ emotional exhaustion leading them to lower turnover intention.

## 1. Introduction

Employee turnover has been extensively researched in organizations. Managers are preoccupied with this issue as it leads to low performance, increases training expenses, replaces employees, and endures lower productivity [1,2,3,4,5]. Turnover relates to employees’ intention to leave a certain organization after a significant period of time [6]. It has been observed that these employees go through a period of reflection before reaching the final decision to leave [7]. In addition, the authors of [8] discovered that 25% of turnover intentions culminated in a definitive turnover. The authors of [9] described turnover intention as the intention of the organizational members to leave their current work and seek other employment prospects due to a certain dissatisfaction with their existing position. Nonetheless, data from [10] show that in the presence of an ethical leader, an organization’s work unit is associated with lower turnover intention.

Leadership studies are becoming increasingly noteworthy in the twenty-first century, as western corporations have brought ethical concerns to the forefront [5,10]. The authors of [11] postulate the need to address the question of how leadership influences employees’ behavioral outcomes. According to a rising body of research on leaders’ personal qualities, conscientiousness was found to be a strong predictor of ethical leadership conduct [12,13,14]. This was found to have a significant impact on employee work satisfaction and loyalty, which further affects employee behavioral outcomes [15,16,17]. While these studies have produced fascinating findings, there is still limited research in providing a clearer understanding of the psychological factors associated with the establishment of ethical leadership and employee behavioral outcomes (i.e., turnover intention). By implying a link between a leader’s conscientiousness and the behavioral pattern required to be seen as an ethical leader, this article investigates whether the trait of conscientiousness may be particularly well suited to explaining ethical leadership and reduced turnover intention among employees.

**Figure 1:**
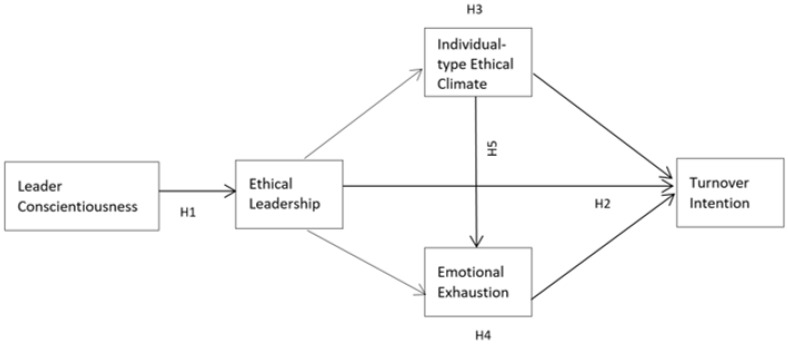
Theoretical model.

Earlier studies demonstrated management failure by displaying poor leadership qualities [18,19,20]. However, negative organizational outcomes such as employees’ turnover intention have received less attention in the context of ethical leadership as a facilitating element [21] and it is necessary to further explore the individual-level ethical climate and emotional exhaustion as its mediating variables. Emotional exhaustion has been widely researched among hospital staff nurses [22], with limited research conducted in manufacturing industries. Studies show that employees who become emotionally exhausted and display high turnover intention from excessive stress induced by the workplace climate have often been linked to poor psychological health [15,23,24]. Relying on [5]’s conceptualization of ethical leadership, the purpose of this study is to advance the understanding of when and why employees decide to withdraw from an organization and further develops and tests a model in which the individual-level ethical climate and emotional exhaustion are positioned as the mediating components influencing turnover intention (shown in Figure 1).

To explain how ethical leadership may inspire and sustain a positive ethical work climate, this study is based on social learning [25] and social exchange [26] theories. The aim of this study concentrates on adding knowledge to the gap on two counts: Does leader conscientiousness serve as the best trait for ethical leadership and mold the workplace ethical climate? Does the presence of an ethical leader imply diminishing emotional exhaustion caused by the workplace leading to reduced turnover intention among the employees working in manufacturing industries? First, while identifying and emphasizing conscientiousness as a prerequisite for ethical leadership is a vital step [12], this study goes further by investigating whether conscientious leaders who act ethically lead to improved job outcomes. Although earlier research linking ethical leaders’ conscientiousness to turnover intention has been found to be limited, the current study contends that the influencing linkages between conscientiousness as a trait and ethical leadership in relation to the outcome (turnover intention) are significant.

Second, this study includes organizational situational factors such as an individual-level ethical climate consisting of a self-interested ethical climate, an ethical climate of friendship, an ethical climate of personal morality, and employees’ emotional exhaustion, in accordance with the social exchange theory [26] to gain a broader understanding of the level of turnover intention. This response calls on recent research [16,27] by focusing on the effect of being ethical while performing a leadership role and its impact on the ethical climate of the work unit even after consistently remaining ethical. More importantly, while answering the call for further investigation regarding turnover intention [28,29], this study delves further by providing insights on adapting an individual-level ethical climate in the work unit and its influence on employees’ emotional exhaustion level. The research also seeks to add to the expanding body of evidence that ethical leadership influences employees’ individual-level behavior [30,31] by creating an impact on their emotional exhaustion and behavioral outcomes (turnover intention). The study tests these relationships based on subordinates’ perspective ratings of leaders’ personalities and organizations’ climate behavior and seeks to add to the literature by proposing ethical leadership as the most effective leadership for the organization and followers.

### 1.1. Leader Conscientiousness and Ethical Leadership

Leadership styles have been repeatedly linked to improvements in work-environment quality and employee retention. Among the Big Five personality traits, conscientiousness has long been considered an important moral assessment [32,33] to predict individual personality. The authors of [32] define one’s conscience as a foundation for moral judgment. Conscientious leaders foster performance by assisting in the development of norms and behaviors that eventually inspire active participation in the improvement of work processes [34]. The author of [35] identifies a conscientious personality as one involving goal-setting, carefulness, dependability, self-discipline, thoroughness, responsibility, deliberation, and persistence. However, research explains that the underlying psychological processes of ethical leaders’ positive influence are still scarce [36].

Ethical leaders display effective leadership skills by introducing a sense of commitment and engagement among employees in the workplace, allowing people to have a more positive attitudes about their jobs [21]. These leaders exhibit “normatively proper behaviors” [10], such as openness and honesty, dependability, and truthfulness—all of which are qualities of conscientiousness [14]. For example, they identified a moderate association between leader conscientiousness traits and ethical leadership in which subordinates evaluate them as ethical leaders. The authors of [13] and [29] found that highly conscientious leaders are likely to be viewed as role models of appropriate behavior provided they act dutifully themselves. Additionally, the author of [12] states that it is a reflecting internal process that instructs the leader to demonstrate ethical behavior and to advocate and promote such behavior in the organization. Furthermore, leaders are viewed as being flexible in their ethical principles when employees respond better by becoming more involved in the organization and minimizing negative outcomes [12]. However, the author of [37] suggests that high consciousness in leaders may lead to adverse impacts because they are seen to have a strong orientation towards goal achievement.

These trends suggest the argument about whether conscientious leaders are likely to develop more attention and be considerate about regularizing aspects of conduct in their daily lives is mixed. Given the correlations between leader conscientiousness and ethical leadership behavior, the study expects to find that hiring or training conscientious leaders will encourage leaders to pay attention to moral issues and that such traits among leaders play an important role in explaining the relationship between ethical leadership and employee work outcome (turnover intention). Thus, according to the above argument, the following hypothesis is proposed:

**Hypothesis** **1 (H1).** *Leader conscientiousness will be positively associated with ethical leadership.*

### 1.2. Ethical Leadership and Employee Turnover Intention

Employee turnover intention is the likelihood of actual turnover caused by inconsistency between employees’ motivation and the organization’s working conditions. The authors of [38] define turnover intention as the willingness to voluntarily leave one’s organization. As a result, a number of studies have been conducted to deepen the understanding of turnover intention in order to address employee retention and enhance organizational effectiveness [39]. Previous research [10] demonstrates the association between an employee and their manager as one of the most critical positions which have shown a significant impact on employee attitudes and behavior results. The authors of [40] point out that when managers implement coercive tactics on their employees, it is expected that employees incline towards withdrawal. To address this condition, ethical leadership researchers demonstrated its positive effect on employees’ behavior outcomes [12,14,28,29,31]. Ethical leadership is described as “the demonstration of normatively performed behavior through two-way personal acts and interpersonal relations” [5].

Grounded on social exchange theory [26], the present study expects to extend a previous study [21] by identifying leaders coordinating their employees’ behavioral reactions in which employers may anticipate their employees’ withdrawal decision if they are expecting a source of support from the organizations’ ethical climate [41,42,43] which could act as a cue to anticipate employees’ decisions. The authors of [44] found that leaders’ actions are ethical when they engage in behaviors which benefit employees, while refraining from acts and behaviors that may harm them. It has also been observed that a leader’s ethical adoption in the work unit helps employees to willingly report ethical challenges (e.g., the ethical climate) that may be worked out with the leader, hence decreasing employee withdrawal intention [45]. Furthermore, the authors of [28] argued that the influence of ethical leadership behavior spreads through the work context and contributes to shaping the organizational members’ commitments towards a lower turnover intention. The main effect of ethical leadership has been investigated in this study because ethical leadership not only motivates employees to engage in their work but can also reduce turnover intention by enhancing the positive strength of work among employees. As a result, from the above evidence, ethical leadership will have an effect on reducing employees’ turnover intention:

**Hypothesis** **2 (H2).** *Ethical leadership will be negatively associated with turnover intention.*

### 1.3. The Mediating Effect of the Individual-Type Ethical Climate

This study is based on social learning theory [25], where ethical leaders are predicted to deliver ethical direction to their employees. These employees imitate such behaviors by observing their leaders’ role-model actions, decisions, and subsequent consequences. This is due to the fact that ethical leaders integrate ethical concepts with leadership skills and place a high priority on accomplishment in order to promote ethical actions that contribute to their organization’s achievement [10,13,46,47,48]. It has been stated that in an organization, ethical leadership becomes more visible to followers when they are seen to set laws and processes and develop behavioral expectations towards a structured ethical climate [5,49].

The organizational climate is one of the most important predictors of individual attitudes and behavioral outcomes found among banking-sector employees [21], and it was also evident in a study of salespeople who were found to be actively involved and devoted to the organization’s goals [11]. To achieve such an ethical climate, the researchers posit that leaders must first set ethical guidelines by rewarding or punishing ethical behavior. Ethical leaders appear to be critical towards adopting an ethical climate because when leaders display ethical behavior, employees are more likely to follow ethical expectations [50]. Individual attitudes and behaviors, however, are significantly influenced by how employees perceive their company’s atmosphere and react with job issues [51]. As a result, corporations are supposed to endeavor to instill ideals of respect, consideration, and professionalism through communicating their corporate values by establishing a moral and ethical climate [52].

The authors of [53] defined an ethical climate as the “prevailing perceptions of normal organizational activities and procedures with ethical meaning.” Researchers previously discovered that a poor ethical climate is associated with negative outcomes (i.e., turnover intention) [54,55,56]. For example, recent studies contend that an organizational climate builds expectations by continuously communicating standards in which leaders affect employees’ observations of the ethical climate toward guidelines and activities [57]. The authors of [58] emphasized that employee’s long-term commitment to a firm is frequently influenced by the ethical climate, which determines whether an employee decides to stay or leave the organization. According to [59], ethical leaders tend to foster a friendly and pleasant workplace in which they assume that subordinates are determined to achieve their best and that supporting their efforts is preferable to directing them. Thus, the following has been advanced by [51]: an individual’s (employee) observations and actions are greatly influenced by how they assume their organizational climate to be positive, which in turn helps them handle their work challenges and decreases turnover intention. However, the authors of [47] argue that leadership actions and outcomes are more visible in a specific organizational climate.

In this study, only three types of ethical climates from the taxonomy [53] were chosen to advance the understanding of individual-type ethical climates (i.e., self-interest—egoistic, friendship—benevolent, and personal morality—principled moral). This study explores employees’ individual perceptions since it concentrates on the exchange interactions between individual employees and their managers. Thus, the ethical environment of the organization acts as a source of normative belief, causing employees to direct their behavior in response to cues from peers and managers. As a result, the study attempts to claim that employees who work under ethical leadership are less likely to be subjected to a bad work environment because ethical leaders are more likely to foster a strong ethical climate and behavioral outcomes. The following hypotheses are proposed based on previous theoretical reasoning:

**Hypothesis** **3 (H3a).** *Ethical leadership will be positively associated with individual-type ethical climate (self-interest, friendship, and personal morality ethical climates)*.

**Hypothesis** **3 (H3b).** *The individual-type ethical climate (self-interest, friendship, and personal morality ethical climates) will be negatively associated with turnover intention*.

**Hypothesis** **3 (H3c).** *The individual-type ethical climate (self-interest, friendship, and personal morality ethical climates) will mediate the relationship between ethical leadership and turnover intention*.

### 1.4. The Mediating Effect of Emotional Exhaustion

Emotional exhaustion (i.e., feeling tired and stressed), the basic characteristic of burnout [60], is not only detrimental for employees’ physical and emotional health, but also negative for business growth. Previously, [60] has stated that while employees are emotionally exhausted, they are no longer able to “give themselves at a psychological level”. This is because when employees face a lack of psychological resources, they are likely to feel vulnerable and unfavorable about their workplace surroundings [61], increasing their desire to leave. Recent studies [16,62], have demonstrated employees suffering from psychologically demanding work appear to be one of the primary drivers of turnover intention. Furthermore, they mention that work intensity is the primary source of stress that contributes to job dissatisfaction, which is directly linked to emotional exhaustion. Employees who have adequate resources are inclined to devote themselves to their work with emotional fortitude and personal resources (e.g., energy and mental efforts), which minimizes the risk of the intention to leave [63].

Furthermore, social exchange theory [26], confirms that employees are driven to return the fair treatment they receive from their ethical leaders [5]. Research has shown that an ethical leader influences the workforce by shaping the work climate, imparting employee with positive feelings [13,64]. Given that the basic feature of ethical leadership is the delivery of moral cues, it is logical to claim that the consequences of ethical leadership are dependent on followers’ moral attention [65]. Additionally, [29] suggest that the ethical behaviors of leaders are critical in promoting productive, value-driven actions among subordinates. Such leaders plan, organize, coordinate, explain, manage, and regulate their subordinates’ activities, thus creating a psychological structure for subordinates. Ethical leaders are thought to instill in their employees an attitude of commitment to the group as a whole, motivating individuals to perform their tasks effectively. As a result, the study concludes that ethical leadership strengthens social exchange mechanisms, hence reducing the direct association between the ethical climate and turnover intention. For the aforementioned reasons, the study proposes the following hypothesis:

**Hypothesis** **4 (H4a).** *Ethical leadership will be negatively associated with employees’ emotional exhaustion*.

**Hypothesis** **4 (H4b).** *Employees’ emotional exhaustion will be positively associated with turnover intention*.

**Hypothesis** **4 (H4c).** *Emotional exhaustion will mediate the negative relationship between ethical leadership and turnover intention*.

**Hypothesis** **5 (H5).** *The individual-type ethical climate will be negatively associated with emotional exhaustion*.

In summary, we propose a mediation model, as shown in Figure 1.

## 2. Materials and Methods

### 2.1. Participants and Procedures

The data for this research were collected through survey questionnaires of subordinates working as executives who are reporting to mid-level managers. In order to get an adequate number of valid samples, a total of 380 questionnaires was sent to nine industrial manufacturing SMEs of Selangor, Malaysia within the period of January to February 2022, from which only 260 questionnaires were usable for the analysis. No missing variables were reported. According to [66], the applicable sample size for structural equation modelling (SEM) is at least 200 and is optimally 400. The study relied on a snowballing sampling method [67] where the survey packages were handed to the human resource managers of manufacturing organizations, who were further requested to reach the right employees with the required criteria (i.e., employees working under mid-level managers in an SME manufacturing organization). Following this, the respondent was asked to provide the same information to his/her co-worker regarding the research project. In addition, the contact person was briefed with an introductory letter that employees who agreed to participate in the surveys had to be subordinates of the same supervisor for at least one year and that the data they submitted were solely for research purposes. Later, surveys were collected from the contact personnel on an appointed date.

The study used a quantitative method to investigate the correlations between the variables. The measurement and structural models were evaluated using partial least squares structural equation modelling (Smart PLS v.3.3.6) software. According to [68], PLS-SEM is a non-parametric, multivariate method for estimating path connections with latent variables. The study is exploratory in nature, using Malaysian manufacturing SMEs as its primary focus. The data for the study were gathered from the manufacturing SMEs, which were members of the Federation of Malaysian Manufacturers [69], and the “the unit of analysis” for this research are the employees working in SME manufacturing organizations.

### 2.2. Measures

The questionnaire in this study followed well-established scales in English. Questionnaires were in English because the intended respondents were employees from various backgrounds and well educated. Prior to the formal data collection, a pilot survey was conducted with employees from manufacturing SMEs. No uncertainty or misunderstanding was reported in the questionnaire.

Leader conscientiousness: A four-item scale was used from the Mini International Personality Item Pool to assess leader conscientiousness [70]. All items were measured on a 5-point Likert scale (1 = strongly disagree; 5 = strongly agree). A sample item here was as follows: “My manager gets chores done right away”. The Cronbach’s alpha was 0.61.

Ethical Leadership: The ethical leadership scale used in this study as the independent variable was adopted from a ten-item scale [10], and the Cronbach’s alpha was 0.88. A five-point Likert scale was used (1 = strongly disagree; 5 = strongly agree). A sample item here was as follows: “My supervisor listens to what employees have to say”. 

Turnover Intention: The dependent variable is turnover intention, which is measured using a three-item scale adapted from [71]. Participants were asked to rate themselves on a 5-point Likert scale (1 = strongly disagree; 5 = strongly agree). A sample item was: “I plan on leaving my job within the next year”. The Cronbach’s alpha was 0.81.

Individual-level ethical climate: The individual-level ethical climate scale [72] is a nine-item scale that is being studied as a mediating variable. The scale originally contained nine items related to the individual-level ethical climate; however, two items were removed from the study in order to meet the reliability and validity requirements in the Malaysian context. All items were scored on a 5-point Likert scale (1 = mostly false; 5 = mostly true). The Cronbach’s alpha was 0.74.

Emotional Exhaustion: The four-item scale developed by [73] was used to assess employees’ emotional exhaustion. Respondents were required to assess the items using a 5-point Likert scale (1 = Never; 5 = Always). A sample item here was: “In this company, working all day is really a strain for me”. The Cronbach alpha for this scale was 0.80.

### 2.3. Statistical Analysis

Initially, the study examined the sample’s demographic information as well as the internal consistency of the constructs. The study contained thirty items, of which five were removed due to the poor threshold value of 0.60, leaving 25 items. The study has assessed the appropriateness of the factor analysis for these 25 items by employing the Kaiser–Meyer–Olkin (KMO) test and Bartlett’s test of sphericity. The overall sampling adequacy was ensured by the KMO test, which showed a result of 0.73 (>0.50), followed by Bartlett’s test, which provided support for the appropriateness of the factor analysis, which showed a result of 3640.041, df 300, and significance at *p* < 0.01. The principal component analysis of the 25 items allowed them to be categorized into seven groups with eigenvalues larger than one: 6.631, 2.990, 2.251, 1.833, 1.604, 1.185, and 1.090. While using self-reported questionnaires to collect data, the common method variance was taken into account because the results of obtained from both the dependent and independent variables were attained from the same individual [74]. Further, to eliminate method biases induced by commonality, the study implemented scale endpoints and anchoring effects. Alternative scale endpoints and formats were used for the predictor and criterion measures. Additionally, Harman’s single factor test was employed as a statistical technique in this study, with all significant constructs included into a principal component factor analysis with 23.90% of variance explained, which is less than 50% [75,76]. Following this, the inter-correlations showed no value of 0.9 or greater, with the maximum inter-correlation being only 0.642 as per Table 1. As a result of both tests, method bias is not a serious concern in our study.

## 3. Results

### 3.1. Participant Characteristics

A total of 260 of the 310 participants completed valid questionnaires, yielding 83%, with males (40.4%) and females (59.6%). The employees’ average age ranged from 35 to 49 years old (41.2%). The highest reported education level was bachelor’s degree (30.0%), while 40.4% of employees had worked under their manager for more than a year, as shown in Table 2.

### 3.2. Description Statistics and Correlations

The research looks into the direct links between leaders’ conscientiousness and ethical leadership, as well as the mediating relationships between ethical leadership, the individual-level ethical climate, emotional exhaustion, and turnover intention. The study has followed [66]’s suggestion that constructs be evaluated to assess their internal consistency reliability, convergent validity, and discriminant validity. The results meet the internal consistency, with composite reliability (CR) scores ranging from 0.745 to 0.902, which meets the suggested threshold of >0.70. While the lowest loadings were discovered to be EC4 (0.559), EC5 (0.446), EC6 (0.597), EC7 (0.475), and EL5 (0.528), out of the 25 total indicators, the highest loading was determined to be ToI1 (0.891). Despite having lower indicator loadings than the criterion, the average variance extracted (AVE) of individual-level ethical climate (EC) exceeds the minimum level of >0.50. As a result, the indicators were retained [68]. Convergent validity was demonstrated by AVE values ranging from 0.595 to 0.720 (Table 3).

The discriminant validity (the degree of divergence of items among constructs or the measure of individual concepts) of this study was evaluated using the heterotrait–monotrait (HTMT) ratio criteria proposed by [77] as shown in Table 4. This criterion was chosen over the Fornell–Larcker criterion because of the criticisms addressed against the latter. The authors of [78] argue that the Fornell–Larcker criterion fails to effectively depict the absence of discriminant validity in common research contexts. As a result, the discriminant validity of this study was determined utilizing the HTMT correlation ratio where none of the values are more than the HTMT limit of 0.85.

### 3.3. Structural Model Analysis

The study described by [68] proposed a distinct evaluation criterion for the structural model. The early stage of the collinearity problem was investigated by measuring the *R*^2^, beta (*β*), and associated *t*-values, followed by the bootstrapping process with a resample of 5000. Furthermore, the study has also considered the suggested predictive relevance (*Q*^2^) and the effect sizes (*f*^2^). However, [79] reported that the *p*-value determines only the existence of the effect, but it does not reveal how large the effect is.

#### Hypothesis Testing

The results in Table 5 reveal that ethical leadership was found to be a strong, significant predictor of leader conscientiousness (*β* = 0.353, *t* = 6.416, *p <* 0.001). Thus, H1 was supported. Similarly, ethical leadership is perceived as having a significant negative impact on employees’ intention to leave (*β* = −0.366, *t* = 8.882, *p <* 0.001). Based on the size of the path coefficient between ethical leadership and turnover intention, it can be argued that ethical leadership has a stronger negative influence on turnover intention, supporting the H2. The authors of [68] suggested that researchers examine the mediating effects of the mediation hypothesis (indirect effect) using the technique of [80,81]. The indirect effect (mediation effect) of individual-level ethical climate H3 has been tested, with results (*β* = −0.095, *t* = 2.423, *p* < 0.008). The analysis found that ethical leadership has a significant positive impact on the workplace’s individual-level ethical climate while diminishing the withdrawal tendency (*β* = 0.525, *t* = 13.326, *p* < 0.001). While ethical leaders’ actions and consequences are more evident in specific organizational climate situations, ethical leaders’ actions and consequences are more noticeable in general. Hence, H3a, H3b, and H3c were supported. In H4, emotional exhaustion is shown to mediate the relationship between ethical leadership and employee turnover intention, which has been found to support the study (*β* = −0.142, *t* = 5.470, *p* < 0.001). This is due to the fact that an ethical leader is thought to have a positive impact on ethical climates such as self-interest, friendship, and personal morality, which leads to a lower withdrawal intention among employees. Thus, H4a, H4b, and H4c were supported. Furthermore, H5 projected that the individual-level ethical climate will be negatively linked with emotional exhaustion (*β* = −0.397, *t* = 2.291, *p* < 0.011). The findings posit that in the presence of a positive ethical climate (self-interest, friendship, personal morality), employees are adapted to lower emotional exhaustion. Hence, H5 was supported.

The study needed to address the collinearity issue with regard to a structural model. In accordance with [68], a multi-collinearity problem is discovered when the biggest VIF is more than 5. However, no indication of significant multicollinearity (Table 5) was discovered among the exogenous factors in the investigation. The VIF values were all less than 5, ranging from 1.247 to 1.446, indicating that the differences discovered in the external constructions and those reported in the endogenous construct were not the same.

The predictive importance of the suggested research model, as well as the use of the blindfolding method, were evaluated. The authors of [68] suggested that by utilizing the blindfolding method only on endogenous constructs, where the *Q*^2^ if found was greater than zero, has predictive relevance for a certain endogenous component [68,82]. As per Table 5, the *Q*^2^ value was (higher than 0), indicating that the suggested model had appropriate predictive significance. Moreover, *Q*^2^ values of 0.35 (large), 0.15 (medium), and 0.02 (small) have been used as relative measures of predictive relevance [68] demonstrating the study’s contention that the endogenous construct had a high predictive significance.

Furthermore, a test on the change in the *R*^2^ value has been performed in terms of the effect size *f*^2^, to determine if an exogenous latent construct has a weak, moderate, or considerable effect on an endogenous latent construct [68,83]. As shown in Table 5, the *f*^2^ had a considerable effect size.

## 4. Discussion

### 4.1. Theoretical Contributions

The current study found it beneficial to investigate approaches to minimize industrial manufacturing employees’ turnover intention. First, while previous research has linked ethical leadership practices to leader conscientiousness [13,14], this research further complements the line of research of [12] by investigating leaders’ conscientiousness as a potentially advantageous personality attribute predicting ethical leadership and one linked indirectly with manufacturing sector employees’ turnover intention. Based on the social learning viewpoint, by emphasizing the critical role of morality in this process, the findings indicate the psychological mechanism that enables conscientious leaders to be more likely to demonstrate ethical leadership actions. According to the hypothesized model, it has been found that leaders with higher conscientiousness quality are morally inclined toward their employees’ needs and subsequently bring positive change in the work unit. Contrary to [37], the result did not find that leader consciousness played any significant negative role in employees’ experiences with respect to turnover intention. Thus, conscientious leaders with a feeling of obligation may make individuals more willing to attempt to do the right thing, and by performing faithfully, conscientious leaders can serve as role models for their subordinates.

Second, the present research contributes to the literature by examining the individual-level ethical climate (self-interest, friendliness, and personal morality) as a situational mediator of ethical leadership and employee turnover intention that has been previously overlooked. In line with [15,29,41], the study found that the individual-level ethical climate plays a positive situational role by directly impacting employees’ emotions and changes in their withdrawal intention. Employees can replicate the behaviors of ethical leaders who establish a fair workplace, convey standards, and support ethical conduct, making employees more dedicated and willing to stay with the firm. As in the study depicted in [27], employees were seen to willingly report to their leader while experiencing organizational adjustment and performed better during the engagement. Furthermore, emphasizing how ethical actions by leaders influence employees’ behaviors according to social learning theory, the employees working under ethical leadership reported stability.

Third, the study examined the emotional exhaustion of employees working in manufacturing organizations to measure the mediating role of emotional exhaustion with the association between ethical leadership and turnover intention. The results were found to be similar to those [84]—that ethical leadership has a significant negative effect on employees’ emotional exhaustion and turnover intention. Furthermore, the results also extend the previous study of [21], which showed emotional exhaustion is lowered in the presence of ethical leadership and that it retains an indirect influence on positive ethical climate in the organization. Employees believe they play a vital role in the business, and ethical leaders who actively involve their subordinates in the organizational process are more likely to profit from higher engagement. Moreover, [15] mentions that organizations can reduce turnover intention by mitigating the detrimental consequences of leaders. They want to work for a leader who upholds high ethical standards and expects the same from his or her employees. As noted by [5], frequent contact between employees and the leader has a good effect on their organizational behavior, which is also supported by [28], which argues that organizations need to pay close attention to the selection and promotion of their managers in order to create a strong ethical climate for the manufacturing industry. On the basis of social exchange theory, the study revealed that in presence of an ethical leader, the subordinate is found to show a positive behavioral effect (lower emotional exhaustion). Furthermore, the current study advanced previous research [16] by focusing on leaders’ influence on employees’ turnover intentions and found an indirect significant negative relation to employee turnover intention. Furthermore, the findings also support the previously stated negative impact of ethical leadership on emotional exhaustion [84].

### 4.2. Practical Implications

The current study’s findings demonstrate that ethical leadership is an effective approach to minimizing turnover intention. It also provides significant practical knowledge that can help and guide the establishment of ethical leadership in firms by diminishing and eliminating manufacturing employees’ turnover intention. Given the linkages among leader conscientiousness, ethical leadership behavior, and turnover intention, the findings suggest that recruiting merely conscientious leaders who are cognitively driven to demonstrate ethical behaviors is insufficient. Rather, encouraging the leaders to pay attention to climatic moral issues in the organizational work unit is also an important consideration. Organizations can use ethical leaders’ influence to improve their employees’ emotional behavior by creating a fair and trustworthy environment and conveying established normative standards. Moreover, the study indicates that leaders having the freedom to make decisions also strengthens the organizations’ ethical climate, which helps to build an environment in which employees feel they belong. For example, ethical leaders should continue to promote an individual-level ethical climate and take efforts to connect their reward and punishment systems with strong ethical expectations, resulting in a stronger, more influential ethical climate in organization.

This study is practically significant because it demonstrates the authority for ethics training in business schools as well as at all levels of the organization. If organizations want to enhance their ethical climate and retention, they should contemplate developing training programs on issues such as stress management, anger management, positive thinking programs, goal definition, and team responsibilities. However, ethical leaders can guide organizations and management to enhance employee well-being and foster healthy workplaces by recognizing and cultivating the tendencies that provide emotional and workplace resources. Thus, emotional exhaustion in employees can be decreased by providing training programs that enable supervisors to convey clear messages to the employees. The potential usefulness of discovering and selecting conscientious leaders, on the other hand, may be lessened because such leaders require suitable employment conditions in order to enhance their ability and further develop and model ethical leadership.

### 4.3. Limitations and Directions for Future Research

While the study has a number of strengths, it is not without limitations. First, the study was conducted in the manufacturing industry in Malaysia. Although the study does not expect the findings to be unique to any specific culture, the use of a Malaysian sample may shed some doubt on its generalizability to other cultures or other contexts and sectors. The findings regarding the relationship between ethical leadership and turnover intention across industries provides an important avenue for future research to investigate such a relationship in greater depth, where the role of the working climate is critical for employees’ response to turnover intention. For this reason, it is thought important to replicate the study’s approach in various contexts and sectors, particularly inside a single organization, as advocated by [11]. Furthermore, cross-cultural research could help to establish generalizations across cultures and reveal whether differences in relationship impacts are attributable to cultural differences or organizational policies and practices.

The study’s second limitation is that there was little research available for some of the links between ethical leadership and situational factors (the individual-level ethical climate). For example, an organizational ethical climate consists of the cross-tabulation of the two dimensions resulting in nine theoretical ethical climate levels [53,85]. Only the individual ethical climate (ethical criterion and locus of analysis) was taken into account in this study, with the individual-level ethical climate found to be a mediator in the relationship between ethical leadership and employee turnover intention, and the unit of analysis being employees’ individual perceptions about their ethical climate in manufacturing organizations. The research was unable to further examine the interaction effects of another most important personality trait—agreeableness—as specified in [5] to this current model. As a result, it is essential to discover and appreciate potential indicators that predict employee turnover intention.

The third limitation is the cross-sectional nature of this study, which lacked experimental design. The researchers have opted for snowball sampling because of the difficulty in conducting a field experiment. The outcome of the analysis was only reported from the employee’s perspective—for instance, the variable of turnover intention was self-reported by the employees and also collected on the same questionnaire. Future research should try to replicate and extend this work utilizing different data sources and non-self-reported turnover indicators, such as actual turnover data [86,87]. Moreover, the study is demonstrated on underlying theories (i.e., social learning and social exchange theories) that account for the effect of turnover intention by analyzing the involvement of two relevant variables as mediators (i.e., individual-level ethical climate and emotional exhaustion). Furthermore, by integrating the individual-level ethical climate with the most researched topics in the field, i.e., emotional exhaustion, to assess the effect on turnover intention, this study broadens the understanding of the role of ethical leadership. In addition, the perspective of this study has been focused on manufacturing-industry employees and the role of ethical leadership and ethical climate (individual-level) in diminishing the emotional exhaustion and turnover intention.

The fourth limitation of this study is that it considered only one dimension of burnout, i.e., emotional exhaustion. In addition, the study has examined the link between ethical leadership and employees’ emotional exhaustion while studying the antecedent trait (leader’s consciousness). Although previous research has looked at the antecedents of a leader’s ethical conduct, additional research is needed to find the traits that would lead to more positive ethical behavior in an organization while also strengthening the connection between leaders and their subordinates [54]. Other variables, such as employees’ educational level or their ethnicity, may have an impact on their level of emotional exhaustion and turnover intention. The results found a significant negative impact on the emotional exhaustion which led to reduced turnover intention. More studies are needed in future to cross-validate our findings using samples drawn from different cultures.

## 5. Conclusions

This study provides a new insight on the condition that leads conscientiousness to be considered an important personality trait, demonstrating a significant positive relationship between leader conscientiousness and ethical leadership. In line with social learning theory, such a trait is essential as a building block toward ethical leadership, instilling a sense of dedication and engagement in employees in the workplace. Furthermore, our research reveals the importance of an individual-level ethical climate as a mediator that has been observed to be more effective in the presence of ethical leadership. Such managers, who demonstrate ethical leadership conduct, have an important influence on the establishment an ethical climate, leading to reduced turnover intention among employees. This study underlines that ethical leadership conduct is related to perceptions of an ethical climate and its influence on employees’ emotional exhaustion, which contributes to it being an essential factor to understanding employees’ turnover intention. It is worth mentioning that organizational leaders must ensure that an individual-level ethical climate is present in the workplace, which has been found to be considerably detrimental towards employees’ emotional exhaustion and withdrawal intention.

## Figures and Tables

**Table 1:** Discriminant validity.

Constructs	1	2	3	4	5
Emotional Exhaustion	**0.782**				
Ethical Leadership	−0.278	**0.715**			
Individual-type Ethical Climate	−0.185	0.642	**0.629**		
Leader Conscientiousness	−0.061	0.369	0.387	**0.772**	
Turnover Intention	0.444	−0.299	−0.299	−0.04	**0.848**

Values on the diagonal (bolded) are square root of the AVE while the off-diagonals are correlations.

**Table 2:** Demographic profile of the study (*n* = 260).

Variable	Categories	Percentage	Mean	Std. Deviation
Gender	Male	40.4	1.60	0.492
	Female	59.6		
Age	Below 25	24.6	1.23	0.863
	26 to 34	30.8		
	35 to 49	41.2		
	50 above	03.5		
Highest education level	High School	10.4	1.89	1.064
	Diploma	26.9		
	Degree	30.0		
	Master	28.5		
	Doctorate	04.2		
No. of years under present manager	2 years or less	40.2	0.82	0.839
	2 to 4 years	40.4		
	5 to 7 years	13.8		
	8 or more	04.6		

**Table 3:** Reliability and convergent validity of study constructs.

Variables	Items	Loadings	CR	AVE
Leader Conscientiousness	CO1	0.832	0.745	0.601
	CO3	0.706		
Emotional Exhaustion	EE1	0.767	0.861	0.611
	EE2	0.886		
	EE3	0.822		
	EE4	0.629		
Individual-type Ethical Climate	EC1	0.613	0.814	0.602
	EC4	0.559		
	EC5	0.446		
	EC6	0.597		
	EC7	0.475		
	EC8	0.786		
	EC9	0.826		
Ethical Leadership	EL1	0.799	0.902	0.711
	EL3	0.631		
	EL4	0.803		
	EL5	0.528		
	EL6	0.719		
	EL7	0.846		
	EL8	0.708		
	EL9	0.656		
	EL10	0.686		
Turnover Intention	ToI1	0.891	0.885	0.720
	ToI2	0.800		
	ToI3	0.851		

**Table 4:** Discriminant validity based on heterotrait–monotrait (HTMT) criterion.

**Constructs**	**1**	**2**	**3**	**4**	**5**
Emotional Exhaustion					
Ethical Leadership	0.279				
Individual-type Ethical Climate	0.348	0.610			
Leader Conscientiousness	0.344	0.667	0.826		
Turnover Intention	0.600	0.342	0.408	0.249	

Shaded boxes are the standard reporting format for HTMT procedure.

**Table 5:** Structural model assessment.

Hypotheses	Relationship	Std. Beta	*t*-Value	*p*-Value	Decision	*R* ^2^	*f* ^2^	*Q* ^2^	VIF
H1	Leader Conscientiousness → Ethical Leadership	0.392	8.743	0.001	Supported	0.138	0.157	0.062	1.247
H2	Ethical Leadership → Turnover Intention	−0.366	7.665	0.001	Supported	0..325	0.008	0.168	1.411
H3a	Ethical Leadership → Individual-type Ethical Climate	0.548	14.378	0.001	Supported	0.268	0.415	0.110	
H3b	Individual-type Ethical Climate → Turnover Intention	−0.351	7.812	0.001	Supported	0.325	0.029		1.446
H3c	Ethical Leadership → Individual-type Ethical Climate → Turnover Intention	−0.095	2.423	0.008	Supported				
H4a	Ethical Leadership → Emotional Exhaustion	−0.351	9.084	0.001	Supported	0.079	0.049	0.037	
H4b	Emotional Exhaustion → Turnover Intention	0.441	8.469	0.001	Supported	0.325	0.183		1.047
H4c	Ethical Leadership → Emotional Exhaustion → Turnover Intention	−0.142	5.470	0.000	Supported				
H5	Individual-type Ethical Climate → Emotional Exhaustion	−0.397	2.277	0.011	Supported	0.079	0.005		

## Data Availability

Available on request from Ms. Saleh, email: tazneenafn@gmail.com.
